# Case Report: A diagnostic chameleon of EBV-associated immune dysregulation: HLH unmasking multiple myeloma with subsequent emergence of aggressive B-cell lymphoma

**DOI:** 10.3389/fimmu.2026.1829220

**Published:** 2026-06-04

**Authors:** Yang Dai, Luocheng Zhang, Xushu Zhong, Jinbing Zhu, Ailin Zhao

**Affiliations:** 1 Department of Hematology, Institute of Hematology, and Center for High Altitude Medicine, West China Hospital, Sichuan University, Chengdu, China; 2 State Key Laboratory of Biotherapy, Collaborative Innovation Center of Biotherapy, West China Hospital, Sichuan University, Chengdu, China; 3 National Facility for Translational Medicine (Sichuan), West China Hospital, Sichuan University, Chengdu, China; 4 West China Medical School, West China Hospital, Sichuan University, Chengdu, China

**Keywords:** B cell lymphoma, Epstein-Barr virus (EBV), hemophagocytic lymphohistiocytosis (HLH), multiple myeloma, R-CHOP

## Abstract

Hemophagocytic lymphohistiocytosis (HLH) is a life-threatening hyper-inflammatory syndrome most commonly triggered by infection, autoimmune disease, or malignancy. Multiple myeloma (MM)-associated HLH is rare. We report a 70-year-old female who presented with HLH as the initial manifestation of MM. Following MM-directed therapy, both MM and HLH achieved remission; however, HLH relapsed with persistent Epstein–Barr virus (EBV) viremia despite continued MM control. Anti-PD-1 monoclonal antibody therapy transiently suppressed EBV viremia, but HLH subsequently recurred. Repeat bone marrow examination revealed an unclassifiable aggressive large B-cell lymphoma. Because tissue biopsy was not feasible, lymphoma classification relied primarily on bone marrow findings, representing an important diagnostic limitation. Although EBER positivity was detected, this finding alone was insufficient to establish a direct pathogenic role of EBV in lymphoma development. Subsequent R-CHOP chemotherapy achieved temporary HLH control; however, severe chemotherapy-related myelosuppression and sepsis developed, and the patient ultimately died of multi-organ failure. This case highlights the challenges of developing lymphoid neoplasms in the setting of EBV-associated immune dysregulation, the diagnostic limitations of bone marrow-based lymphoma classification, the transient and limited efficacy of PD-1 blockade for EBV reactivation control, and the therapeutic difficulties encountered in this highly complex clinical setting.

## Background

Hemophagocytic lymphohistiocytosis (HLH) is a life-threatening hyperinflammatory syndrome characterized by uncontrolled immune activation and cytokine storm (1, 2). In adults, secondary HLH is predominantly caused by infections, autoimmune diseases, as well as malignancies, particularly lymphoid neoplasms (3). Among infectious triggers, Epstein–Barr virus (EBV) accounts for the vast majority (4).

Nevertheless, Multiple myeloma (MM) is only rarely reported as a precipitating factor for HLH; moreover, the sequential emergence of distinct lymphoid neoplasms under EBV-associated immune dysregulation remains poorly characterized (5). Here, we report an unprecedented and singular case of secondary HLH occurring at the onset of newly diagnosed MM with concomitant high EBV viral load, followed by relapse heralding the emergence of aggressive B-cell lymphoma (6).

## Case presentation

A 70-year-old woman presented with ten days of high-grade fever (maximum 39.8 °C) and progressive cytopenias. On admission, she was febrile and weak, without overt lymphadenopathy. Laboratory evaluation revealed pancytopenia: hemoglobin 79g/L (115-150g/L), platelets 40×10^9^/L (100-300×10^9^/L), white blood cell count 2.13×10^9^/L (3.5-9.5×10^9^/L), neutrophils 1.43×10^9^/L (1.8-6.3×10^9^/L), ferritin 9928 (24-336ng/mL), soluble IL-2 receptor (sCD25): 37587 (223-710u/mL). PCT 0.75(<0.046ng/mL). Comprehensive investigations for cytomegalovirus (CMV) and influenza virus were negative, as were blood culture results. Chest CT revealed no obvious infection images. Abdominal CT revealed an enlarged spleen. The patient presented with fever persisting over one week, two lineage cytopenia, markedly elevated ferritin and soluble CD25 levels, and splenomegaly, fulfilling 5 of 8 HLH-2004 diagnostic criteria and establishing a diagnosis of HLH (**Table 1**). Genetic screening for HLH-associated mutations yielded no pathogenic variants.

**Table 1:** HLH diagnostic criteria fulfilled at three episodes.

HLH-2004 diagnostic criteria		The first HLH (MM stage)	The second HLH	The third HLH (lymphoma stage)
Fever (>38.5 °C)		Yes (Maximum 39.8 °C; fever persisted >1 week)	Yes (Maximum 39.5 °C; fever persisted >1 week)	Yes (Maximum 38.5 °C; fever persisted >1 week)
Splenomegaly		Yes (on abdominal CT)	Yes (on abdominal CT)	Yes (on abdominal CT)
Cytopenias (Affecting >=2 lineages)	Hemoglobin (<90 g/L)	Yes (79 g/L)	Yes (88 g/L)	Yes (63 g/L)
	Platelet (<100×10^9^/L)	Yes (40×10^9^/L)	Yes (16×10^9^/L)	Yes (2×10^9^/L)
	Neutrophils (<1.0×10^9^/L)	No (1.43×10^9^/L)	Yes (0.49×10^9^/L)	Yes (0.59×10^9^/L)
Hypertriglyceridemia (Triglycerides >=3.0 mmol/L or >=265 mg/dL) or Hypofibrinogenemia (Fibrinogen <=1.5 g/L)		No	No	No
Ferritin (>=500 ng/mL)		Yes (9,928 ng/mL)	Yes (13,980 ng/mL)	Yes (22,736 ng/mL)
sCD25 (>=2,400 U/mL)		Yes (37,587 U/mL)	Yes (7,487 U/mL)	Yes (24,890 U/mL)
Decreased or absent natural killer (NK) cell activity		NA	NA	NA
Hemophagocytosis (bone marrow, spleen, or lymph nodes)		Yes (Bone marrow)	NA	No

**Figure 1:**
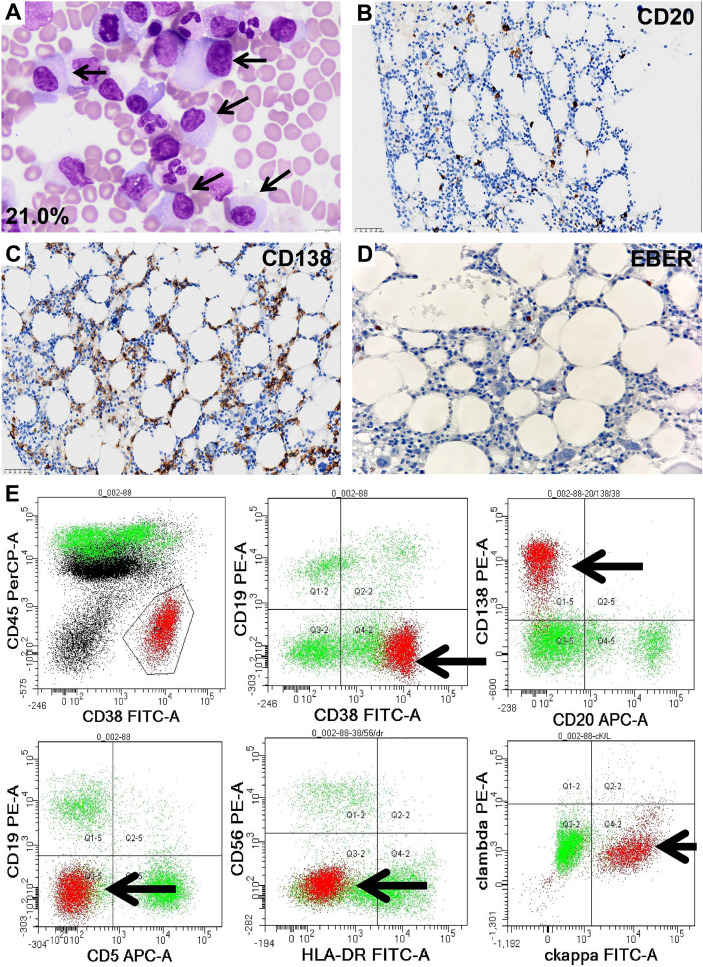
Initial bone marrow findings (MM stage). **(A)** Bone marrow aspirate smear showing increased plasma cells. (Wright–Giemsa stain, ×1000). **(B)** CD20 immunohistochemical staining (× 40). **(C)** CD138 immunohistochemical staining confirming plasma cell infiltration (× 40). **(D)** EBER in situ hybridization highlights scattered medium-sized EBER-positive cells (× 400). **(E)** Flow cytometry demonstrating monoclonal plasma cells. Black arrows indicate abnormal plasma cells.

Etoposide plus dexamethasone (ED) was urgently administered for HLH control. Concurrently, a comprehensive workup to identify the underlying etiology was performed, revealing markedly elevated EBV-DNA (1.43 × 10^6^ copies/mL). Autoimmune antibody screening was negative. Bone marrow examination demonstrated increased plasma cells (21%), including 3% immature forms; these plasma cells were relatively large with round, slightly eccentric nuclei, finely dispersed chromatin, and abundant deeply basophilic cytoplasm containing vacuoles; occasional binucleated and dysplastic forms were noted; hemophagocytosis was present. Flow cytometric analysis identified 10.2% abnormal cells expressing CD38, CD138, and cytoplasmic kappa light chain restriction. These cells were negative for CD45, CD19, CD20, CD56, HLA-DR, and cytoplasmic lambda light chain (**Figure 1**). In addition, an IgG-Kappa type monoclonal protein was detected. Albumin 27.6 g/L (40-55), β2-microglobulin 10.5 mg/L (0.7-1.8), LDH 873 IU/L (120-250). Fluorescence *in situ* hybridization (FISH) for multiple myeloma-associated cytogenetic abnormalities revealed 13q14 deletion, 1p32 deletion, and 1q21 amplification. No pathogenic TP53 variants were detected. These results established the diagnosis of MM (R2-ISS Stage III). Treatment with bortezomib and dexamethasone (BD) was administered and rapidly stabilized the patient’s condition, accompanied by improvement in HLH-related inflammatory markers. The patient subsequently received two cycles of the BD regimen, with MM remaining in remission throughout this period.

Three months post-treatment, the patient experienced a second occurrence of HLH (**Table 1**). No monoclonal protein was detected, consistent with complete remission of MM; however, EBV-DNA level was markedly elevated (2.6×10^6^copies/mL). In the absence of evidence for MM progression and with markedly elevated EBV-DNA, recurrent HLH was considered temporally associated with EBV reactivation. Then, ED was administered for two weeks to control HLH, resulting in normalization of temperature, partial hematologic recovery, and significant reductions in ferritin and sCD25 levels, indicating partial response of HLH. Currently, allogeneic hematopoietic stem cell transplantation (allo-HSCT) remains the only curative modality for relapsed/refractory EBV-associated HLH (7). However, the patient’s advanced age and poor performance status rendered her ineligible for allo-HSCT. Our previous pilot study demonstrated the promising efficacy of programmed cell death protein 1 (PD-1) blockade in this clinical setting (8). Accordingly, following a comprehensive discussion with the patient and her family and upon obtaining informed consent, sintilimab (a Chinese-developed PD-1 monoclonal antibody) 100 mg was administered by intravenous infusion. Following infusion, the patient developed recurrent fever with a maximum temperature of 39 °C, which was responsive to celecoxib. Both ferritin and sCD25 levels increased, peaking at 14,440 ng/mL and 4,135 U/mL, respectively on day 6 post-infusion, followed by gradual normalization toward baseline values. This represented a mild HLH flare following PD-1 monoclonal antibody administration; however, the patient achieved spontaneous resolution without specific anti-HLH therapy. The patient was subsequently discharged without fever. Monthly outpatient follow-up for EBV-DNA monitoring demonstrated persistent negative amplification for five consecutive months, accompanied by sintilimab infusions (100 mg once monthly), another three times in total. No further fever or adverse reactions were observed. During this period, PD-1 blockade demonstrated transient virologic suppression and temporary clinical stabilization.

**Figure 2:**
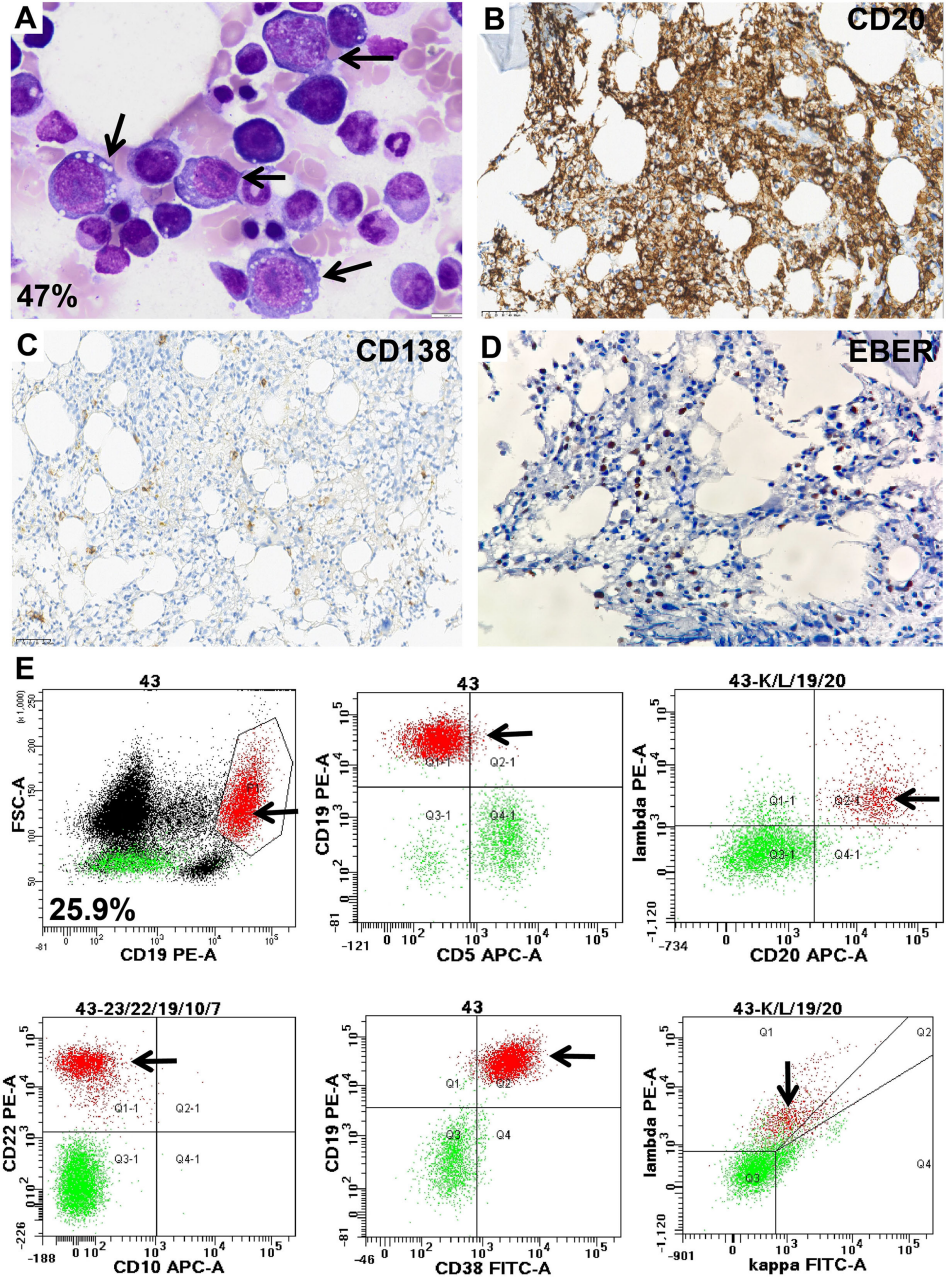
Bone marrow findings at HLH relapse (lymphoma stage). **(A)** Bone marrow aspirate smear showing medium-to-large abnormal lymphoid cells (47% estimated by morphology), present singly or in clusters. Cells exhibited round-to-oval nuclei with folding and vacuolation, strand-like chromatin, and indistinct nucleoli. Cytoplasm was deeply basophilic and frequently vacuolated. (Wright–Giemsa stain, ×1000). **(B)** CD20 staining showing B-cell infiltration (× 40). **(C)** CD138 staining demonstrating decreased plasma cells compared with the initial examination (× 40). **(D)** EBER in situ hybridization highlights medium-sized EBER-positive cells (× 400). **(E)** Flow cytometry analysis revealed 25.9% abnormal lymphoid cells within the gated population, demonstrating high forward scatter properties and expressing CD19, CD20, CD22, CD38, as well as monotypic lambda light chain restriction. Black arrows indicate abnormal lymphoma cells (aggressive B-cell lymphoma cells).

However, a third episode of HLH occurred (**Table 1**), prompting another bone marrow examination, which demonstrated 47% abnormal cells, present singly or in clusters; these cells were medium-to-large with round to oval morphology. Cytoplasmic volume was variable, deeply basophilic, and frequently vacuolated. Nuclei were round to oval with nuclear folding and vacuolation; chromatin was strand-like with indistinct nucleoli. Hemophagocytosis was not observed. Bone marrow flow cytometry revealed 25.9% abnormal lymphoid cells demonstrating high forward scatter (FSC) properties, consistent with large cell size. The abnormal population showed positive expression of CD19, CD20, CD22, CD38, and dim/partial FMC7, with monotypic lambda light chain restriction. The following markers were absent: CD5, CD10, CD23, CD103, CD200, and kappa light chain. Bone marrow biopsy demonstrated that lymphoid cells in small sheets or focal distribution within the bone marrow were positive for CD20 and PAX5, and negative for CD3, CD5, CD138, kappa, lambda, CD56, and TDT, Cyclin D1, CD23, CD34, CD117, and CK. CD30 showed scattered positivity, and CD10 displayed partial positivity. These findings support bone marrow involvement by B-cell lymphoid neoplasm (**Figure 2**). As the patient had no palpable superficial lymphadenopathy and was in poor clinical condition despite splenomegaly, histological examination of tissues other than bone marrow was not feasible. Consequently, the diagnosis relied exclusively on bone marrow findings, representing an important limitation. Although morphologic, immunophenotypic, and clinical features were consistent with an aggressive B-cell lymphoma, a definitive classification could not be established in the absence of tissue biopsy, and the case is therefore best regarded as unclassifiable. Based on the overall clinical presentation and available diagnostic data, a working diagnosis of aggressive B-cell lymphoma was made, and lymphoma-directed therapy was initiated. Given her poor performance status, dose-reduced R-CHOP was selected: rituximab 600 mg on day 1, cyclophosphamide 400 mg on day 2, epirubicin 50 mg on day 3, vindesine 2 mg on day 3, and dexamethasone 10 mg on days 2–6. Following chemotherapy, the patient’s temperature normalized and hemophagocytic markers improved significantly: ferritin decreased from 10,552 to 3,028 ng/mL, and sCD25 declined from 33,150 to 4,490 U/mL. However, 2 weeks later, the patient developed recurrent fever without evident signs of infection. HLH markers rose significantly again, attributed to disease progression. A second dose-reduced R-CHOP was administered (rituximab 600 mg on day 1, cyclophosphamide 800 mg on day 2, epirubicin 50 mg on day 2, vindesine 2 mg on day 2, and dexamethasone 10 mg on days 2–6), resulting in temperature normalization and improvement in HLH parameters. However, pancytopenia persisted with remarkable neutropenia. Granulocyte colony-stimulating factor (G-CSF) was administered for leukocyte recovery. Thirteen days later, the patient developed abdominal pain, diarrhea, and vomiting following consumption of contaminated food, accompanied by fever. Intestinal infection was suspected; imipenem-cilastatin was promptly initiated, and blood cultures were obtained. While no organisms were isolated. Despite intensive antimicrobial therapy, fever and symptoms remained poorly controlled. The patient ultimately succumbed to septic shock two days later.

## Discussion

**Figure 3:**
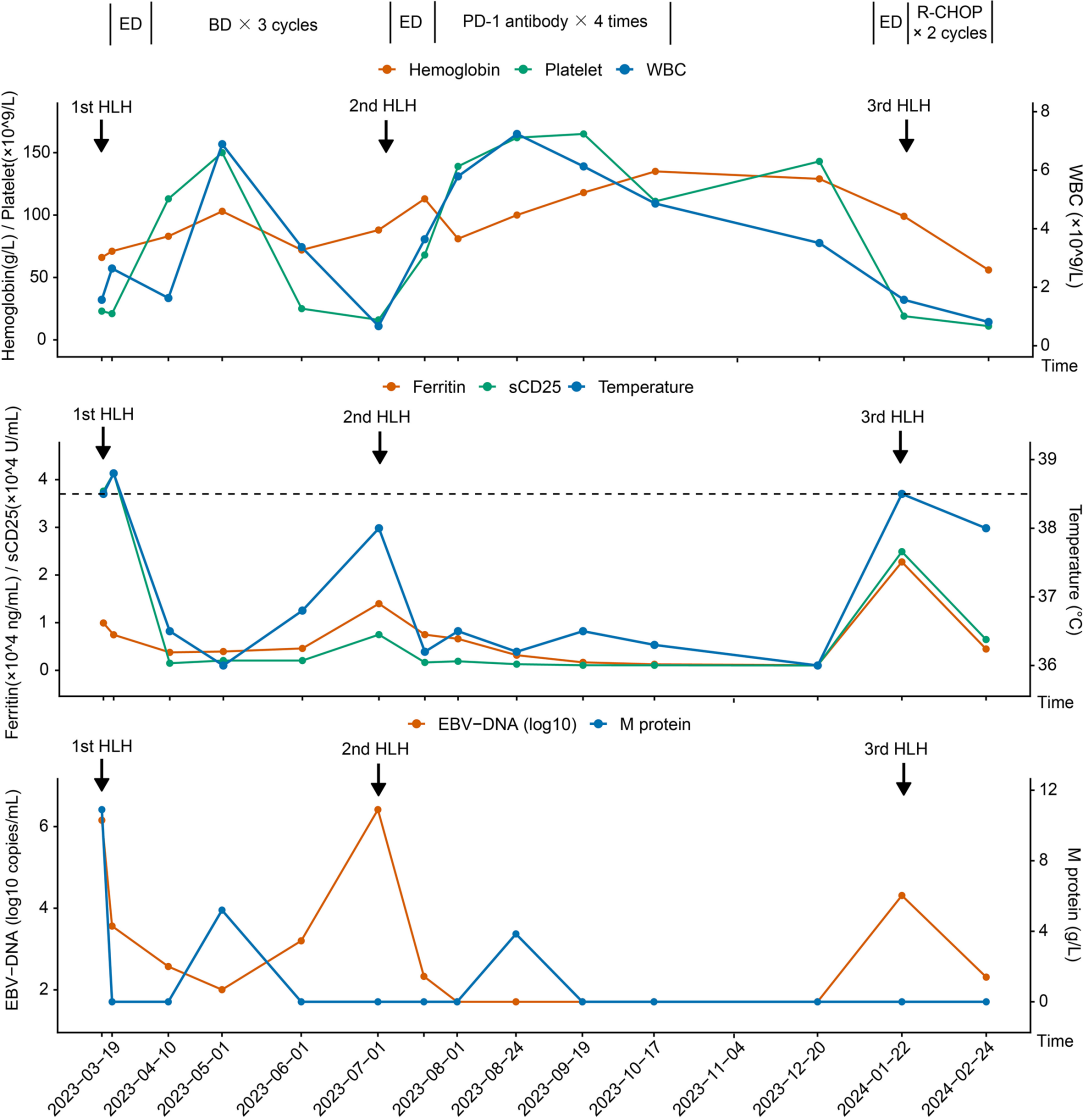
The patient's treatment progress over time and changes in laboratory indicators E: etoposide; D: dexamethasone; B: bortezomib; R-CHOP: rituximab plus cyclophosphamide, epirubicin, vindesine and prednisone; M protein: monoclonal protein; sCD25 (IL-2R): soluble interleukin-2 receptor; WBC: white blood cell.

This case captures the sequential emergence of three immunologically distinct lymphoid malignancies—MM, EBV-associated lymphoproliferation, and aggressive B-cell lymphoma—unified by HLH presentation in a single patient (**Figure 3**). Following the diagnosis of MM, the patient received MM-directed therapy with resultant HLH remission. The second relapse of HLH prompted aggressive bone marrow evaluation, leading to the diagnosis of aggressive B-cell lymphoma, unclassifiable. Lymphoma-specific treatment stabilized the disease once again. It underscores that HLH is a multifactorial syndrome requiring treatment of the underlying etiology. However, the possible mechanisms driving this complex sequence—MM onset, EBV-associated lymphoproliferation, and subsequent emergence of B-cell lymphoma within a single patient—remain poorly understood. Moreover, treatment approaches in such a complex scenario also require exploration.

### Multiple myeloma and immune dysregulation

MM is a hematologic malignancy characterized by the clonal proliferation of plasma cells (9). It induces significant immune suppression through a variety of mechanisms, including T-cell exhaustion, cytokine dysregulation, and the secretion of immunosuppressive factors such as IL-6, IL-10, and TGF-β. These factors create an immunosuppressive microenvironment that predisposes patients to infections, including EBV reactivation, and increases the risk of developing secondary malignancies like EBV-associated lymphoma (10). In this patient, both MM and its treatment likely contributed to an immunocompromised state, thereby predisposing to EBV reactivation, which in turn may have triggered HLH. The genetic abnormalities observed in this patient, such as deletions in RB1, CDKN2C, and the gain/amplification of CKS1B, further underscore the complexity of MM-related immune dysregulation and its role in predisposing the patient to both infections and secondary malignancies (11).

### Possible relationship of EBV and aggressive B-cell lymphoma

EBV was detected during the disease course and may have been associated with the HLH episode; however, its relationship to the subsequent development of aggressive B-cell lymphoma remains unclear. Although EBV can infect B cells in the setting of immune dysregulation, the presence of EBER positivity alone does not demonstrate involvement of EBV in the malignant clone. In this context, EBV viremia is more likely to reflect underlying immune dysfunction related to MM or its treatment rather than indicating a direct pathogenic role (12).

A temporal association was observed between EBV reactivation and the later diagnosis of aggressive B-cell lymphoma, unclassifiable; however, this observation does not establish causality. Importantly, the possibility that lymphoma was already present but remained undetected earlier in the disease course should be strongly considered as an alternative explanation. In this context, the lymphoma may have occurred subclinically and only became clinically apparent during the third HLH episode. At that time, bone marrow examination revealed abnormal lymphoid cells consistent with an aggressive B-cell lymphoma, unclassifiable.

Taken together, these findings highlight the importance of ongoing surveillance in patients with HLH, particularly in the context of persistent immune dysregulation. EBV detection in this setting should be interpreted cautiously, as it may represent an epiphenomenon rather than a mechanistic driver of disease progression (13, 14).

### Challenges with immunotherapy: the failure of PD-1 monoclonal antibody

The treatment of this patient was complicated by underlying immune dysregulation related to both MM and EBV reactivation. Despite initial improvements with HLH-directed therapy, including the ED regimen, and anti-myeloma therapy with the BD regimen, the patient subsequently experienced disease relapse. This reflects a key challenge in managing patients with MM and EBV-associated complications, namely persistent immune suppression. MM-related T-cell exhaustion, together with elevated immunosuppressive cytokines, may have facilitated EBV immune evasion and reduced the effectiveness of subsequent therapies, including immune checkpoint inhibition. We observed that PD-1 blockade was associated with an initial decrease in EBV-DNA levels (8). Given the single-case nature and the lack of standardized response assessment, these findings should be interpreted with caution, and no firm conclusions regarding treatment efficacy can be drawn. However, this initial virologic response did not translate into durable disease control, and lymphoma progression continued. This observation highlights the potential limitations of PD-1 inhibition in the setting of significant immune dysregulation (15). Mechanistically, the immunosuppressive tumor microenvironment in EBV-related lymphoma, potentially involving increased PD-L1 expression and recruitment of regulatory T cells (Tregs), may further impair immune recognition and attenuate the effectiveness of immune checkpoint inhibitors (16).

### Management complexity and the evolving role of combination therapeutics

This case underscores the challenges of managing HLH-onset B-cell lymphoma in the context of MM and EBV reactivation. The patient’s course illustrates the limitations of single-agent therapy, including PD-1 inhibition, and highlights the need for a coordinated, multimodal approach. Combining chemotherapy (e.g., R-CHOP) with antiviral agents such as ganciclovir or valganciclovir, alongside immune checkpoint inhibitors when appropriate, allows simultaneous targeting of the tumor, viral reactivation, and underlying immune dysregulation. Close monitoring of EBV-DNA levels further supports timely therapeutic adjustments. Despite these strategies, the patient’s poor outcome emphasizes the high complexity and fragile prognosis in such overlapping pathophysiological conditions.

## Conclusion

This case illuminates the immunological heterogeneity underlying HLH, tracing the dynamic interplay between EBV-associated immune dysregulation, MM, and aggressive B-cell lymphoma within a single patient. It underscores the critical importance of maintaining vigilance for occult malignancies and infectious triggers both at HLH onset and during subsequent relapses. Furthermore, this case exemplifies the therapeutic challenges intrinsic to malignancy-associated HLH, emphasizing the necessity of multimodal combination strategies that concurrently address the hyperinflammatory syndrome and its evolving underlying cause.

## Data Availability

The original contributions presented in the study are included in the article/supplementary material. Further inquiries can be directed to the corresponding author.
